# A purified MAA-based ELISA is a useful tool for determining anti-MAA antibody titer with high sensitivity

**DOI:** 10.1371/journal.pone.0172172

**Published:** 2017-02-21

**Authors:** Takasumi Shimomoto, Leonard B. Collins, Xianwen Yi, Darcy W. Holley, Zhenfa Zhang, Xu Tian, Koji Uchida, Chunguang Wang, Sohvi Hörkkö, Monte S. Willis, Avram Gold, Scott J. Bultman, Jun Nakamura

**Affiliations:** 1 Department of Environmental Sciences and Engineering, University of North Carolina, Chapel Hill, North Carolina, United States of America; 2 Lineberger Comprehensive Cancer Center, University of North Carolina, Chapel Hill, North Carolina, United States of America; 3 Department of Genetics, University of North Carolina, Chapel Hill, North Carolina, United States of America; 4 School of Bioagricultural Sciences, Nagoya University, Nagoya, Japan; 5 Medical Microbiology and Immunology, Research Unit of Biomedicine, Faculty of Medicine, University of Oulu, Oulu, Finland; 6 Medical Research Center and Nordlab Oulu, University Hospital and University of Oulu, Oulu, Finland; 7 Department of Pathology & Laboratory Medicine, University of North Carolina, Chapel Hill, North Carolina, United States of America; 8 McAllister Heart Institute, University of North Carolina, Chapel Hill, North Carolina, United States of America; Monash University, AUSTRALIA

## Abstract

Atherosclerosis is widely accepted to be a chronic inflammatory disease, and the immunological response to the accumulation of LDL is believed to play a critical role in the development of this disease. 1,4-Dihydropyridine-type MAA-adducted LDL has been implicated in atherosclerosis. Here, we have demonstrated that pure MAA-modified residues can be chemically conjugated to large proteins without by-product contamination. Using this pure antigen, we established a purified MAA-ELISA, with which a marked increase in anti-MAA antibody titer was determined at a very early stage of atherosclerosis in 3-month *ApoE*^*-/-*^ mice fed with a normal diet. Our methods of N^ε^-MAA-L-lysine purification and purified antigen-based ELISA will be easily applicable for biomarker-based detection of early stage atherosclerosis in patients, as well as for the development of an adduct-specific Liquid Chromatography/Mass Spectrometry-based quantification of physiological and pathological levels of MAA.

## Introduction

**Fig 1:**
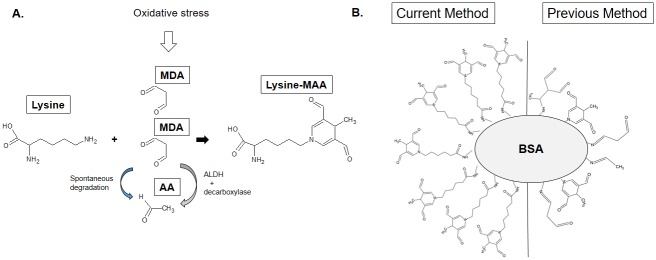
Structure of the MAA-lysine adduct and pMAA and crMAA epitopes. (**A**) 1,4-dihydropyridine-type MAA-lysine adducts are formed by a reaction between acetaldehyde and two equivalents of MDA with a primary amine, usually at the ε-position amino moiety of a lysine residue on the target protein. (**B**) BSA chemically conjugated to purified MAA-6ACA (MAA-lysine analog) was used in the present study and compared to BSA attached to crMAA epitopes, which were utilized in many previous reports.

Lipid peroxidation produces a wide variety of reactive aldehydes, which can form covalent adducts with proteins [1]. These protein adducts can initiate pro-inflammatory responses, and the resulting inflammation caused by these aldehyde-derived protein adducts has been implicated in chronic inflammatory diseases, such as atherosclerosis [2]. During the development of atherosclerosis, protein adducts can be generated by MDA and its degradation product acetaldehyde, which are lipid peroxidation products reactive towards lysine residues on proteins (Fig 1A) [3]. Specifically, 1,4-dihydropyridine-type MAA-modified LDL, which is a form of oxidized LDL (oxLDL), has been implicated in atherogenesis [3–5]. MAA-lysine adducts have been reported to be highly stable [6, 7], toxic [8], pro-inflammatory [9], and profibrogenic [10, 11]. Repeated immunization with MAA-modified protein induces robust antibody production even in the absence of adjuvant [12]. Thus, MAA-lysine adducts have been proposed to be one of the most potent atherogenic protein adducts caused by lipid peroxidation [3, 4]. As such, MAA adducts appear to play a critical role in atherogenesis [3].

Studies have found that serum antibodies against MAA-modified proteins are associated with active and chronic stages of atherosclerosis in humans [13] and that there are detectable levels of anti-MAA antibody even during the development and progression of atherosclerosis [13–15]. These studies have detected the anti-MAA antibody using ELISA plates coated with antigens that are reported to be predominantly a 4-methyl-1,4-dihydropyridine-3,5-dicarbaldehyde derivative of an amino group of protein carriers [16]. However, given the number of lysines found throughout the carriers used in the studies, this cyclic fluorescent adduct was likely not the only product present. For example, a 1:1:1 adduct without fluorescent properties has been reported to be present within the antigen mixture [17]. The heterogeneity of the MAA epitopes, in addition to the other adducts generated by the reaction of MDA and acetaldehyde, likely affect the specificity and sensitivity of these anti-MAA assays. Therefore a method of generating homogeneous MAA-adducted proteins to assay for MAA adducts is important for early diagnosis of atherosclerosis.

Published MAA adduct preparations involve reaction of acetaldehyde and two equivalents of MDA with a primary amine, usually the ε-amino group of a lysine residue on the target protein [18]. During this reaction, many stable (e.g. 2:1:1 product) and unstable adducts (e.g. MDA-lysine) are generated (Fig 1B) [7, 18, 19]. However for early and accurate detection and diagnosis of atherosclerosis, improved sensitivity and specificity of diagnostic biomarker assays is imperative. Thus, in the present study, we synthesized pMAA-lysine and pMAA-6ACA, an MAA-lysine analog. The purified MAA adducts were conjugated through the carboxylic acid moiety to the amino groups of BSA or KLH by the EDC crosslinking reaction (Fig 1B and S1A Fig). Using the purified antigens, we tested the immunogenicity of pMAA molecules and analyzed the serum titer of the anti-MAA-lysine antibody in the atherosclerotic *ApoE*^*-/-*^ mice, for the first time in the absence of confounding factors such as contaminating epitope by-products of the reaction with MDA and acetaldehyde. The pMAA antigen-based ELISA, using BSA chemically conjugated to purified MAA adducts, has not only proven to be more sensitive and specific than the crude MAA antigen-based ELISA that is currently in use but has also been able to detect markedly increased anti-MAA antibody titers in the serum of ApoE^-/-^ mice at a very early stage of atherosclerosis.

## Materials and methods

### Materials

Boc-lysine, 6-ACA, acetaldehyde, BSA, KLH, TFA, TMB, and H_2_O_2_ were purchased from Sigma-Aldrich (St. Louis, MO). Malondialdehyde bis(dimethyl acetal), the Imject EDC mcKLH Spin Kit, goat anti-rabbit IgG (H+L) antibody with HRP, and goat anti-mouse IgM secondary antibody were obtained from Thermo Scientific (Rockford, IL). EnVision+Single Reagents anti-mouse-HRP and rabbit anti-human IgG F(ab’)2 fragment antibody with HRP were purchased from Dako North America, Inc. (Carpentaria, CA) and Jackson Immuno Research Laboratories (West Grove, PA), respectively.

#### Synthesis of *N*^ε^-(MAA)-L-lysine (MAA-lysine) adducts

MDA was generated as previously reported [20]. Briefly, 704 μL of malondialdehyde bis(dimethyl acetal) was hydrolyzed with 96 μL of 4M HCl in the presence of 3.2 mL of water at 37°C for 10 min. The reaction was stopped by adjusting the pH to 7.4 with NaOH. The total volume was brought to 8 mL with water to prepare 500 mM MDA. MAA-lysine was synthesized as previously described for the MAA-BSA preparation [21] with some modifications (S1B Fig). 4 mM of Boc-lysine, 4 mM of acetaldehyde, and 8 mM of MDA were dissolved in water or PBS. The reaction mixture was incubated at 37°C for 3 days. During this time, a yellowish color developed. MAA-Boc-lysine was purified by HPLC using an Agilent 1200 HPLC system (Agilent, Santa Clara, CA) with a Poroshell 120 EC-C18 column (Agilent, 4.6 x 50 mm, 2.7 um) and isocratic elution by 0.1% formic acid and 25% acetonitrile in water at a flow rate of 1 mL/min and UV detection at 264 nm. Fractions (3 mL) were collected by an autosampler, with both the tray and fraction collector chambers maintained at 4°C. The retention time of the fraction containing MAA-Boc-lysine was determined, and a 3.8 to 4.2 min fraction of the mobile phase eluate containing MAA-Boc-lysine was collected automatically. The collected fractions were evaporated and further incubated with 150 μL of 100% TFA overnight to remove the Boc protecting group. Following evaporation, the fractions were dissolved in HPLC-grade water for subsequent HPLC purification. MAA-lysine was purified by the HPLC system described above using a gradient elution program as follows: eluant A, 0.1% formic acid in water; eluant B, acetonitrile, starting at 2% B increasing linearly to 30% B over 2 min and held at 30% B for an additional 3 min before re-equilibration at 2% B for 4 min. The retention time of MAA-lysine was determined, and a 3.8 to 4 min fraction containing MAA-lysine was collected by autosampler. The fractions were evaporated and used for LC-MS and NMR characterization, and BSA/KLH-conjugation.

### Synthesis of MAA-6ACA adducts

MAA-6ACA was synthesized as described for the MAA-lysine preparation with some modifications (S1C Fig). 4 mmoles of 6-ACA, 4 mmoles of acetaldehyde, and 8 mmoles of MDA were dissolved in water or PBS. The reaction mixture was incubated at 37°C for 3 days. MAA-6ACA was purified by HPLC as described above using a gradient elution program as follows: 0.1% formic acid in water (A) and acetonitrile (B) at 2% B linearly increasing to 5% B over 2 min, then increased linearly to 25% B over 0.5 min, held at 25% for an additional 3.5 min, linearly decreased to 2% B over 0.5 min and maintained at 2% B for 3.5 min. The retention time of MAA-6ACA was determined, and a 6.4 to 7.1 min fraction of the eluate containing MAA-6ACA was collected by autosampler. The fractions were evaporated and used for LC-MS, NMR characterization, and BSA/KLH-conjugation.

### LC-MS analysis

MAA-Boc-lysine, MAA-lysine, and MAA-6ACA were characterized with an Agilent Technologies liquid chromatograph-mass spectrometer system consisting of a series 1200 HPLC and 6520 Accurate Mass Q-TOF mass spectrometer (Santa Clara, CA). Products were injected on a Waters Acquity UPLC CSH Fluoro-phenyl column, 2.1 mm x 100 mm, 1.7 μm particle size. Mobile phase was delivered isocratically at 0.2 mL/min using 70% water containing 0.1% formic acid and 30% acetonitrile. Solvent flow was diverted to waste for the first 1.2 min of the analysis. Mass spectrometer parameters were set to the following values: positive ionization mode, capillary voltage of 3500 V, nebulizing gas pressure of 40 psi, drying gas temperature of 300°C, drying gas flow of 12 L/min, and fragmentor voltage of 150 V. Scans from *m/z* 100 to *m/z* 1700 were acquired at a rate of 1 scan/s in the high-resolution, low-mass instrument mode. Reference masses used for real-time mass axis adjustment were purine, *m/z* 121.050873 and HP-0921, *m/z* 922.009798.

### NMR spectrometry

^1^H NMR spectra were recorded on a Varian INOVA 400 spectrometer at 400 MHz in ACN-*d*_3_ or DMSO-*d*_6_, as specified. Chemical shifts are reported in ppm relative to TMS.

#### NMR spectrometry for MAA-Boc-lysine

UV (ddH_2_O): λ_max_ = 260 nm. ESI-MS: *m*/*z* calc for C_19_H_29_N_2_O_6_: 381.2025, obs, 381.2019 MH^+^, *m/z* 325.1397 [MH—Boc] ^+^. ^1^H NMR (400 MHz, DMSO-*d*_6_): 9.23 (singlet, 2H, C*H*O), 7.33 (singlet, 2H, dihydropyridine-*H2*,*H6*), 6.82 (doublet, 1H, *J* = 7.5 Hz, amide), 3.84–3.76 (multiplet, 1H, H^α^), 3.61 (quartet, 2H, *J* = 6.5 Hz, dihydropyridine-*H4*), 3.52 (triplet, 2H, *J* = 6.9 Hz, C^ε^*H*_2_), 1.76–1.52 (multiplet, 4H, C^β,δ^*H*_*2*_), 1.35–1.26 (m, 2H, C^γ^*H*_2_), 1.35 (singlet, 9H, Boc-C*H*_3_), 0.92 (doublet, 3H, *J* = 6.5 Hz, C*H*_3_) ppm.

#### NMR spectrometry for MAA-lysine

C_14_H_20_N_2_O_4_:UV (ddH_2_O): λ_max_ = 260 nm. ESI-MS: *m*/*z* calc for C_14_H_21_N_2_O_4_: 281.1501, obs 281.1498 MH^+^. ^1^H NMR (400 MHz, DMSO-*d*_6_): 9.26 (singlet, 2H, C*H*O), 7.32 (singlet, 2H, dihydropyridine-*H2*,*H6*), 3.62 (quartet, 1H, dihydropyridine-*H4*), 3.50 (triplet, *J* = 6.9 Hz, 2H, C^ε^*H*_2_), 3.17–3.11 (m, 1H, H^α^), 1.51–1.83 (m, 4H, C^β,δ^*H*_*2*_), 1.13–1.37 (m, 2H, C^γ^*H*_2_), 0.92 (doublet, *J* = 6.5 Hz, 3H, C*H*_3_) ppm.

#### NMR spectrometry for MAA-6ACA

C_14_H_19_NO_4_:UV (ddH_2_O): λ_max_ = 260 nm. ESI-MS: *m*/*z* 266.1391 MH^+^. ^1^H NMR (400 MHz, DMSO-*d*_6_): δ 9.22 (singlet, 2H, C*H*O), 7.34 (singlet, 2H, dihydropyridine-*H2*,*H6*), 3.61 (quartet, *J* = 6.5 Hz, 1H, dihydropyridine-*H4*), 3.53 (triplet, 2H, *J* = 7.2 Hz, C6*H*_2_), 2.22 (triplet, 2H, *J* = 7.2 Hz, C2*H*_2_COOH),1.64 (quintet, 2H), *J* = 7.3 Hz, 1.54 (quintet, 2H *J* = 7.5 Hz,), 1.25–1.33 (m, 2H) (C3*H*_2_, C4*H*_2_, C5*H*_2_), 0.92 (doublet, 3H, *J* = 6.5 Hz, CH_3_) ppm.

### Fluorescence measurements

Fluorescent properties of MAA-6ACA were characterized using a CLARIOstar microplate reader (BMG LABTECH) equipped with a scanning mode of continuous adjustable wavelengths (320–850 nm). Fluorescence measurements were also performed for MAA-lysine and MAA-6ACA and their BSA/KLH conjugate complexes using a FLX800 microplate fluorescence reader (Bio-Tek) equipped with excitation (360/40, 400/10, and 485/20) and emission filters (460/40, and 528/20).

### Preparation of antigens (S1A Fig)

50 nmol of MAA-lysine or MAA-6ACA were coupled to either 2 mg of BSA or KLH using the Imject EDC mcKLH Spin Kit, according to the manufacturer’s directions. EDC-mediated amide formation was used for conjugation between MAA epitopes containing a carboxyl moiety and either BSA or KLH. The antigens were purified by spin column and sterile filtered (0.2 μm, Fisher Scientific). Pure antigens were referred to as pMAA-lysine-BSA, pMAA-6ACA-BSA, and pMAA-6ACA-KLH. In order to compare pMAA-lysine-proteins and the crude antigens utilized previously, a reaction with BSA, MDA, and acetaldehyde was performed. BSA (20 mg/7ml in PBS) was incubated with 4 mM acetaldehyde and 8 mM MDA at 37°C for 24 hours. The reaction product was extensively dialyzed for 72 hours in PBS. This crude immunogen was referred to as crMAA-BSA.

### Animal treatment, blood collection, and serum preparation

All mouse experiments were performed using protocols approved by the Institutional Animal Care and Use Committees of UNC and in accordance with federal guidelines. *ApoE*^*-/-*^ mice on a C57BL/6 background and their *wild-type* controls were obtained from The Jackson Laboratory. For addressing the immunogenicity of MAA-lysine, 8-month aged C57BL/6 female mice (3 mice per group) were used. The mice were treated intraperitoneally with BSA or pMAA-lysine-BSA (51 fluorescence units [measured by FLX800 with filter of Ex360/Em460] per 103 μg BSA in 200 μL PBS) once a week for 6 weeks without adjuvant. Seven days after the final injection, mice were euthanized by CO_2_ euthanasia. Blood was collected from the abdominal vein for serum sample collection. Serum samples were stored at -70°C until use. A four-fold serial dilution of each serum sample was prepared using a 5-fold diluted supernatant of 5% Casein PBS suspension. For studying the association between an increase in MAA antibody titer and atherosclerosis, 3-month old C57BL/6 and *ApoE*^*-/-*^ male mice (4 mice per genotype) were utilized for quantitating levels of antibody against MAA. Blood samples were collected from either the maxillary vein or abdominal vein, followed by serum separation. Serum samples were stored at -70°C until use. Each sample was then diluted 320 fold with 1% BSA in PBS for analysis with ELISA.

### ELISA

#### ELISA-based analysis characterizing pMAA-6ACA-BSA using previously-reported anti-MAA antibodies

An indirect ELISA was performed to compare the reactivity of four different previously-reported anti-MAA antibodies [15, 22, 23] against pMAA-6ACA-BSA. 96-well plates (Corning Incorporated, Kennebunk, ME) were coated with pMAA-6ACA-BSA (50 μL/well) at 4°C overnight. After washing, followed by blocking with 3% BSA in PBS, 50 μL of the anti-MAA antibodies were incubated at different concentrations at 4°C overnight. A two-fold serial dilution of each anti-MAA antibody was prepared using 1% BSA in PBS. 1% BSA in PBS was used as a negative control. After washing each well, quantitation of primary antibody binding was performed through the reaction with peroxidase-labeled secondary antibodies. The TMB/H_2_O_2_ substrate was added to all the wells and kept at room temperature for 30 min. The plates were then read with a plate reader (Vmax Kinetic Microplate Reader, Molecular Devices, Sunnyvale, CA) at a wavelength of 650 nm. Each antibody value was corrected by subtracting the OD of the negative control from the value of each sample. The primary and secondary antibodies used for the ELISA were as follows: 1] moMoAb-1F83 [23] and EnVision+Single Reagents anti-mouse-HRP (Code K4001, Dako North America, Inc., Carpentaria, CA); 2] rabPoAb [22] and goat anti-rabbit IgG (H+L) antibody with HRP (Thermo Fisher Scientific); 3] huFull-MoAb (IFUf-08_108) [15] and rabbit anti-human IgG F(ab’)2 fragment antibody with HRP (Jackson Immuno Research Laboratories); and 4] huPre-MoAb (IFUp-08_107) [15] and rabbit anti-human IgG F(ab’)2 fragment antibody with HRP as described above.

#### ELISA for determining anti-MAA antibody titers of serum samples obtained from mice immunized with pMAA-lysine-BSA or BSA

The serum samples of mice immunized with pMAA-lysine-BSA or BSA were separated from the blood to determine the IgG and IgM antibody values against pMAA-6ACA-KLH. Blood samples were collected into 2 mL tubes and centrifuged at 2,000 *g* for 10 min at 4°C. After separation, aliquots of serum were frozen at -80°C until analysis. The samples were diluted and used for ELISA-based analysis. pMAA-6ACA-KLH (50 μL/well with equivalent fluorescence to pMAA-6ACA-BSA) was coated in each well of the 96-well plates at 4°C overnight. After washing, followed by blocking with the supernatant of 5% Casein PBS suspension (hereafter referred to as supernatant A), 50 μL of the serum samples were incubated at different concentrations at 4°C overnight. A four-fold serial dilution of each serum sample was prepared using 5-fold diluted supernatant A. The 5-fold diluted supernatant A was used as a negative control. After washing each well, quantitation of the antibody binding was performed through a reaction with peroxidase-labeled secondary antibodies (IgG: EnVision+Single Reagents anti-mouse-HRP; IgM: Goat anti-Mouse IgM secondary antibody with HRP). The TMB reaction and plate reading were performed as described above.

#### ELISA for quantitating anti-MAA antibody titers of serum samples obtained from wild-type and ApoE^-/-^ mice

The serum anti-MAA IgG and IgM titers in wild-type and *ApoE*^*-/-*^ mice were determined using 96-well plates coated with pMAA-6ACA-BSA or crMAA-BSA. The plates were coated with equivalent amount of fluorescence between two types of antigens. After blocking with 3% BSA in PBS, 50 μL of the 320-fold diluted serum samples in 1% BSA PBS solution were incubated at 4°C overnight. 1% BSA PBS solution was used as a negative control. After washing each well, the quantitation of primary antibody binding was performed as described above.

### Statistical analysis

The antibody values were indicated as mean ± SD of the mean, and the statistical differences between the two groups (*ApoE*^-/-^ vs *wild-type* mice; pMAA-6ACA-BSA plate vs crMAA-BSA plate) were evaluated by unpaired Student t-tests after log transformation. A p-value < 0.05 was considered significant.

## Results

### Synthesis and purification of N^ε^-MAA-lysine

**Fig 2:**
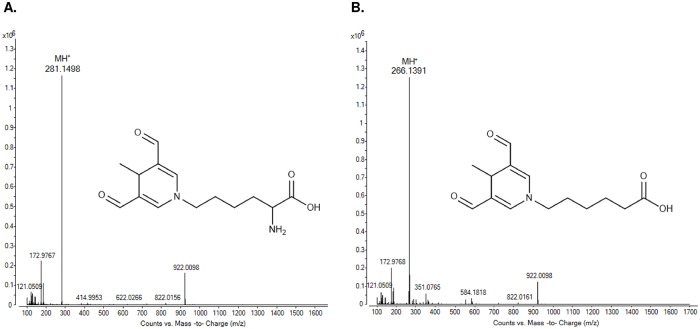
The full scan mass spectrums of MAA-lysine and MAA-6ACA. (**A**) The full scan mass spectrum shows the protonated molecular ion for MAA-lysine at *m/z* 281.1498. Other peaks are minor background ions and reference ions *m/z* 121.0509 and *m/z* 922.0098. (**B**) The full scan mass spectrum shows the protonated molecular ion of MAA-6ACA at *m/z* 266.1391. Other peaks are minor background ions and reference ions *m/z* 121.0509 and *m/z* 922.0098.

The ε amine of lysine is usually the target site for modification by MAA [24]; therefore, N^ε^-(3,5-diformyl-4-methylpyridin-1(4*H*)-yl)lysine was the synthetic target. The Boc-protected lysine adduct was synthesized by incubating Boc-lysine with MDA and acetaldehyde in a 1:2:1 ratio. The procedure for purifying the MAA-lysine is described in detail in S1B Fig. The reaction mixture was analyzed by LC-MS and fractions containing the target product (S2A Fig) were collected with confirmation of the structure by ^1^H NMR (S2B Fig). As anticipated, based on previous reports, multiple contaminating adducts were present. The reaction of the Boc-protected lysine with MDA and acetaldehyde produced unstable adducts (e.g., acetaldehyde-Boc-lysine, MDA-Boc-lysine, and MDA-acetaldehyde (1:1)-Boc-lysine) as well as the stable target. Previous studies on MAA have performed dialysis with the assumption that contaminating protein adducts are unstable and eliminated; however, we confirmed that even extensive dialysis of the mixtures from the direct reaction of BSA, acetaldehyde, and MDA at various concentrations does not remove contaminating protein adducts, as determined by their fluorescence characteristics. Following deprotection of the Boc group, MAA-lysine was purified by HPLC and the peak for pMAA-lysine was identified. The purity and identity of the MAA-lysine adduct was confirmed by ^1^H NMR (S2C Fig) and the molecular composition was determined by exact mass measurement of the molecular ion (Fig 2A).

### Synthesis and purification of N^ε^-MAA-6ACA

As an alternative to the two-step HPLC purification procedure for pMAA-lysine synthesis, MAA-6ACA, an analog of MAA-lysine that does not contain the α-amino group, was synthesized by the reaction of 6-ACA with MDA and acetaldehyde (S1C Fig). pMAA-6ACA was purified by HPLC and its identity and purity were confirmed by LC-MS and ^1^H NMR (Fig 2B and S2D Fig).

### Fluorescence properties of pMAA adducts

The purified adducts exhibited fluorescence, as expected for the 1,4-dihydropyridine chromophore in MAA-lysine [6, 16, 23, 25]. pMAA-6ACA has an excitation/emission maximum of 399/462 nm. These values are in line with previously characterized MAA-lysine analog fluorescent properties [23].

### Anti-MAA antibody reactivity to pMAA-6ACA-BSA

**Fig 3:**
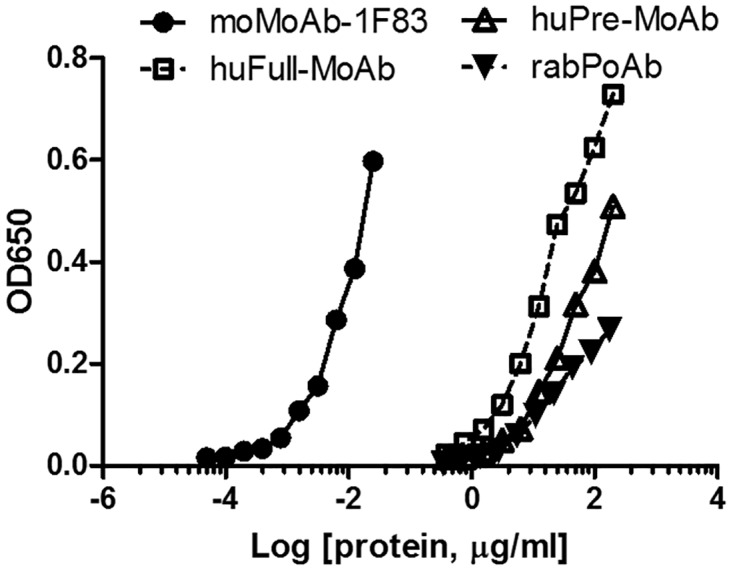
An ELISA method for characterizing the activity of anti-MAA antibodies to pMAA epitopes. Using the ELISA-based assay, we analyzed the four anti-MAA antibodies that were previously used (moMoAb-1F83, huPre-MoAb, huFull-MoAb and rabPoAb). The ELISA plates were first coated with the pMAA-6ACA-BSA antigens. The moMoAb-1F83 reacted with pMAA-6ACA-BSA at ~4 orders of magnitude lower concentration compared to the other anti-MAA antibodies.

By ELISA-based analyses, the four previously-used anti-MAA antibodies we tested recognized the pMAA-6ACA-BSA seeded on the bottom of a 96-well plate, all at different reactivities (Fig 3). The anti-MAA monoclonal mouse antibody (1F83) reacted with the MAA antigen at ~4 orders of magnitude lower concentration compared to the other anti-MAA antibodies.

### Immunogenicity of pMAA-lysine-BSA

**Fig 4:**
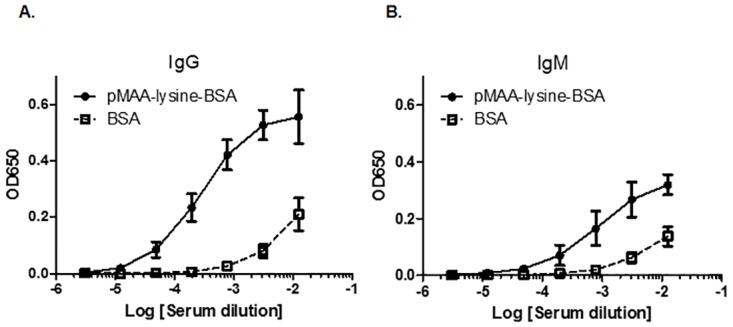
The immunogenicity of pMAA-lysine-BSA in the absence of adjuvant. C57BL/6 mice were injected i.p. with pMAA-lysine-BSA or BSA in the absence of adjuvant. The antibody titers of IgG (**A**) and IgM (**B**) against pMAA-lysine were detected using pMAA-6ACA-KLH-coated plates. The anti-MAA antibody titers were clearly increased in pMAA-lysine-BSA-immunized mice compared to the controls (BSA-treated mice). Error bars represent SD.

The immunogenicity of the MAA unit was previously characterized using a crude MAA-modified BSA mixture (henceforth referred to as crude MAA-BSA, “crMAA-BSA”) in the absence of adjuvant. To evaluate whether pMAA-lysine-BSA also shows immunogenicity in mice, C57BL/6 mice were treated intraperitoneally with pMAA-lysine-BSA or BSA in the absence of adjuvant. The antibody titers of IgG and IgM directed against pMAA-lysine, which were detected using pMAA-6ACA-KLH-coated plates, showed trends in accordance with the dilution levels of the samples. The titers were clearly increased in pMAA-lysine-BSA-immunized mice compared to controls (BSA-treated mice) (Fig 4A and 4B).

### Anti-MAA antibody titer in the serum of atherosclerosis-prone *ApoE*^-/-^ mice

**Fig 5:**
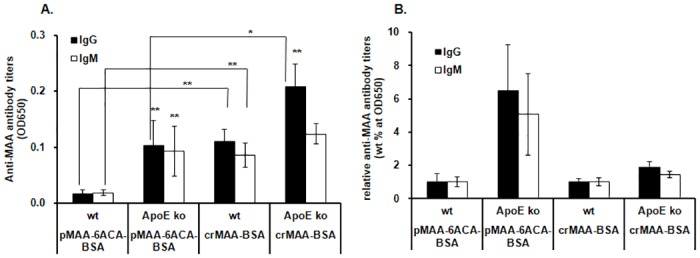
Serum anti-MAA IgG and IgM antibody levels in *wild-type* and *ApoE*^-/-^ mice. **(A)** Serum anti-MAA IgG and IgM antibody levels in wild-type and *ApoE*^-/-^ mice. The anti-MAA IgG and IgM antibody levels showed significant differences between *wild-type* and *ApoE*^*-/-*^ mice with the pMAA-6ACA-BSA and crMAA-BSA ELISAs for all except the IgM levels with crMAA-BSA ELISA (*: p<0.05; **: p<0.01). In addition, the differences between pMAA-6ACA-BSA and crMAA-BSA ELISAs in the *wild type* and *ApoE*^*-/-*^ mice for anti-MAA IgG and IgM antibody levels were significant for all except the IgM levels in *ApoE*^*-/-*^ mice. **(B)** The anti-MAA IgG and IgM antibody titers in *ApoE*^-/-^ mice were normalized to those of *wild-type* mice. The individual titer values of *ApoE*^-/-^ animals were divided by the average titer value of the *wild-type* animals. Of note, the differences between the antibody titers in the *wild-type* and *ApoE*^-/-^ mice were much greater with the pMAA-6ACA-BSA ELISA than with the crMAA-BSA ELISA. Error bars represent SD. Due to the redundancy of the statistical analyses, we did not include any asterisks for statistical significance in Fig 5B.

A previous study reported that oxLDL levels are comparable in the peripheral blood of *wild-type* and *ApoE*^-/-^ mice fed a normal diet at 10 weeks of age but increases and peaks at 20 weeks of age in the *ApoE*^-/-^ mice [26]. To determine whether a pMAA antigen-based ELISA can detect early increases in anti-MAA antibody titers in atherosclerosis, we next addressed whether serum levels of IgG and IgM antibodies against pMAA are increased at a very early stage of atherosclerosis in *ApoE*^-/-^ mice fed with a normal diet. Blood serum was collected from 3-month-old *ApoE*^-/-^ mice and used in an ELISA to detect antibody titers. In addition, the sensitivities of ELISAs were compared between the pMAA-6ACA-BSA- and the crMAA-BSA-coated ELISAs (the plates were coated with antigens containing equivalent amounts of fluorescence). The IgG and IgM antibody values using the pMAA-6ACA-BSA-coated plates in *ApoE*^-/-^ mice were significantly higher than those in *wild-type* mice, with 6.5-fold and 5.1-fold increases, respectively (Fig 5A and 5B). On the other hand, the IgG and IgM antibody values using the crMAA-BSA-coated plates in *ApoE*^-/-^ mice showed only 1.9-fold and 1.4-fold higher values, respectively, compared to the values in *wild-type* mice. ELISAs using serially diluted serum samples (1:80 to 1:1280 dilutions) showed similar results to Fig 5B at different dilutions (S1 Table). These results observed with the pMAA-6ACA-BSA ELISAs indicate that the levels of antibodies against pMAA are markedly increased at very early stages of atherosclerosis. Notably, *wild-type* mice showed 6.9- and 4.8-fold higher IgG and IgM levels, respectively, using the crMAA-BSA-coated plates compared to pMAA-6ACA-BSA-coated plates (Fig 5A). In contrast, the antibody titers in *ApoE*^-/-^ mice using crMAA-BSA-coated plates were only slightly increased compared to pMAA-6ACA-BSA-coated plates.

## Discussion

In this study, we developed a method to purify N^ε^-MAA-L-lysine molecules. Protein adducts were then generated by conjugation with either pMAA-lysine or pMAA-6ACA, free of contamination with modified protein impurities present in the reaction mixtures of MDA and acetaldehyde. This method, for the first time, allows for the investigation of the biological response specific to pMAA-lysine and the antibody response against MAA-lysine in atherosclerosis animal models. The ELISA plates coated with pMAA-6ACA-BSA detected a marked increase in anti-MAA antibody titer in *ApoE*^*-/-*^ mice with higher specificity and sensitivity than the crMAA-BSA-coated ELISA. In contrast to pMAA-6ACA-BSA plates, the *wild-type* mice showed high background IgG and IgM levels using the crMAA-BSA-coated plates. These results suggest that the previous method of extensive dialysis of mixtures from the direct reaction of BSA, acetaldehyde, and MDA was unlikely to remove contaminating protein adducts, which left the confounding factor of antibody responses to antigens other than MAA. Indeed, when we incubated MDA, acetaldehyde and 6-ACA at 37°C followed by HPLC-UV separation, we found that the yield of many major peaks (including MAA-6ACA) were increased, indicating the production of many different, stable adducts. These results suggest that the crMAA-BSA mixture contains many different stable epitopes including MAA. Compared to the current ELISA methods using crMAA-BSA epitope, the pure-MAA ELISA shows improvements in terms specificity and sensitivity and has the potential for detecting early stage atherosclerosis.

Previous studies have demonstrated high titers of IgG antibodies directed against MDA-LDL in 5–6 month old atherosclerosis-prone *ApoE*^*-/-*^ mice fed a diet of regular mouse chow. However, these results were based on a crude MDA-protein-coated radioimmunoassay [27]. Our ELISA technique, using pMAA-6ACA-BSA-coated plates, shows a substantial increase in anti-MAA IgG and IgM antibody levels in *ApoE*^-/-^ mice fed a normal diet, even as early as at three months of age. Further, the differences between the antibody titers in the *wild-type* and *ApoE*^-/-^ mice were much greater with the pMAA-6ACA-BSA ELISA than with the crMAA-BSA assay (Fig 5A and 5B). These results indicate that the high purity of the antigen improves the sensitivity of the assay. These results strongly suggest that the pMAA-BSA ELISA method may be a useful tool to detect early stages of atherosclerosis in patients. In future studies, we hope to apply the pMAA-based ELISA using samples from patients at different stages of atherosclerosis.

In humans, anti-MAA antibodies exist as natural IgM antibodies in the umbilical cord blood of new born babies [15], and anti-MAA IgA, IgM, and IgG antibodies have been detected in the peripheral blood of normal individuals [3, 15, 28]. Such results strongly suggest that MAA epitopes are present under normal physiological conditions and that the total burden of MAA-adducted proteins increase with the progression of atherosclerosis, leading to an increase in anti-MAA antibody generation in atherosclerosis patients. To understand the pathogenesis of the disease, it is crucial to know the kinetics and regions of accumulation of MAA-modified proteins during the development of atherosclerosis. Since it generally takes ~2 weeks to observe a significant increase in antibody titer after a boost vaccination in mice [29, 30], the marked increase in anti-MAA antibody titer in 3-month-old *ApoE*^-/-^ mice in our study suggests that ApoE deficiency may cause an increase in MAA epitopes at as early as 11 weeks of age. Because *ApoE*^-/-^ mice fed with a regular chow diet exhibit only very subtle phenotypes at 10 weeks, with small lesions on 0.5% of the surface of the aorta [26], the 6.5-fold and 5.1-fold increases in IgG and IgM antibodies, respectively, against the pMAA group in *ApoE*^-/-^ mice at 3 months indicates that antibodies against the pMAA group have potential as a very sensitive biomarker for early atherosclerosis. Further studies are necessary to understand the mechanism by which MAA epitopes are increased in *ApoE*^-/-^ mice, as well as the time course of MAA-lysine accumulation. Development of an ultra-sensitive LC-MS-based quantitative analysis using pMAA-lysine standards will be vital to answering these critical questions.

Our method also has substantial potential to increase the specificity of an ELISA-based method for detecting anti-MAA antibody titers. It is noteworthy that there were significant differences in anti-MAA antibody titers with the pMAA-6ACA-BSA and crMAA-BSA ELISAs, particularly in *wild-type* mice (Fig 5A). The IgG and IgM antibody titers in *wild-type* mice using crMAA-BSA-coated plates were significantly higher compared to pMAA-6ACA-BSA-coated plates. These results strongly suggest that using 96-well plates coated with pMAA-6ACA-BSA significantly decreases background resulting from immunoreactive contaminants, such as other adducts present in the MDA–acetaldehyde reaction. These results also suggest that there may be significant levels of MDA- and acetaldehyde-derived antibodies, in addition to anti-MAA antibodies, in mice under normal conditions. The antibodies detected in the *wild-type* mice by the crMAA-BSA ELISA could be derived from both previously identified and unidentified protein adducts, including MDA-acetaldehyde-lysine 1:1:1 adducts, MDA-lysine adducts, and acetaldehyde-lysine adducts [31, 32]. HPLC analysis detected multiple peaks after incubation of MDA, acetaldehyde, and Boc-lysine at physiological temperature and pH. Some of these adducts may be abundant enough to persist even after the crMAA-BSA antigen purification by extensive dialysis. Our results also suggest that, at early stages of atherosclerosis in this mouse model, atherosclerotic conditions may stimulate formation of antibodies specific to MAA-lysine adducts.

In addition to improving ELISA-based analyses, our pMAA antigens may prove to be a great tool to investigate the atheroprotective effects of the immunization of MAA-modified proteins. In animal models, anti-oxLDL antibody (Fab or Fv fragment) infusion and immunization of MDA- or MAA-modified LDL, which leads to high levels of serum antibodies (IgM and possibly IgG1), have both been shown to confer protection against atherosclerosis [4, 33, 34]. These increases in serum antibodies against MDA- and MAA-modified LDL appear to interfere with the interaction between MDA- and MAA-lysine epitopes and macrophages, resulting in inhibition of the inflammatory response. This inhibition is believed to further inhibit both the engulfment of oxLDL by macrophages as well as MAA-mediated inflammation. However, as mentioned previously, the MDA and MAA preparations used in previous studies for immunization resulted in crude, heterogeneous mixtures of various adducts. Since previous studies used impure immunogens for investigating their atheroprotective properties [4, 35], their results need to be interpreted with caution because of a potential increase in non-targeted immune responses caused by the contaminating protein adducts. Therefore, it is important to determine the exact protein adducts that confer atheroprotection by immunization. One of these reports attempted to identify the MDA-derived protein adducts critical for rescue [4]. Interestingly, they found that the responsible adducts could be, in fact, the 1,4-dihydropridine-type MAA-protein adducts based on their immunogenicity potential. Although the authors of this study attempted a more rigorous purification protocol, their efforts still yielded a heterogeneous pool of adducts. These studies further highlight the need for investigations conducted with pMAA adducts as well as the identification of the various contaminating protein adducts generated by reaction with MAA. In our study, BSA was conjugated with pMAA free of contaminating epitopes and we successfully increased anti-MAA antibodies in serum of mice immunized with pMAA-lysine-BSA in the absence of adjuvant. Thus, it will be informative to utilize pMAA-lysine-BSA and pMAA-6ACA-BSA immunogens for improved testing of atheroprotection by MAA immunization.

## Supporting information

S1 Table Ratios of *ApoE*^*-/—*^to-*wild-type* serum antibody titers against pMAA-6ACA-BSA or crMAA-BSA. (DOCX)

S1 Fig Preparation of MAA-lysine, MAA-6ACA, and pMAA-lysine-BSA complex. (DOCX)

S2 Fig The full scan mass spectrum and NMR results of MAA-Boc-lysine and NMR results of MAA-lysine and MAA-6ACA. (DOCX)
