# Supplementary material for: A purified MAA-based ELISA is a useful tool for determining anti-MAA antibody titer with high sensitivity
Source: PLoS One. 2017 Feb 21;12(2):e0172172. doi: 10.1371/journal.pone.0172172 (PMC5319763; doi:10.1371/journal.pone.0172172)
Supplement: S1 Table — (DOCX) [file pone.0172172.s001.docx]

**Table S1. Ratios of *ApoE^-/-^*-to-*wild-type* serum antibody titers against pMAA-6ACA-BSA or crMAA-BSA**

| Serum dilution | pMAA-6ACA-BSA | | crMAA-BSA | |
| --- | --- | --- | --- | --- |
|  | IgG | IgM | IgG | IgM |
| 1:80 dilution | 6.5 | 5.2 | 1.5 | 1.3 |
| 1:320 dilution | 5.6 | 5.3 | 2 | 1.5 |
| 1:1280 dilution | 5.6 | 5 | 2.2 | 1.3 |
