# Supplementary material for: A purified MAA-based ELISA is a useful tool for determining anti-MAA antibody titer with high sensitivity
Source: PLoS One. 2017 Feb 21;12(2):e0172172. doi: 10.1371/journal.pone.0172172 (PMC5319763; doi:10.1371/journal.pone.0172172)
Supplement: S1 Fig — (DOCX) [file pone.0172172.s002.docx]

**Figure S1.**

**A.**

**
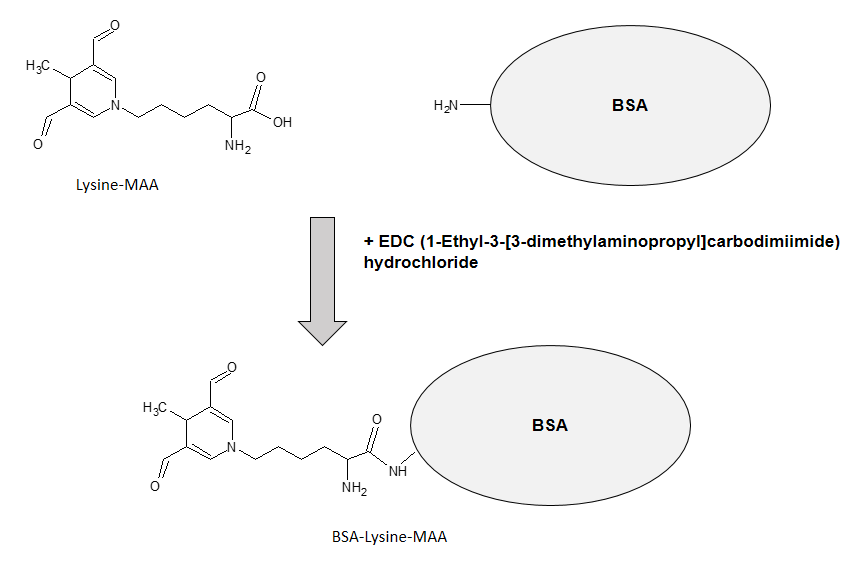
**

Preparation of pMAA-lysine-BSA complex. Pure MAA-lysine was coupled to BSA using the Imject EDC mcKLH Spin Kit. EDC-mediated amide formation was used for conjugation between pure MAA-lysine containing a carboxyl moiety and BSA.

**Figure S1.**

**B.**

**
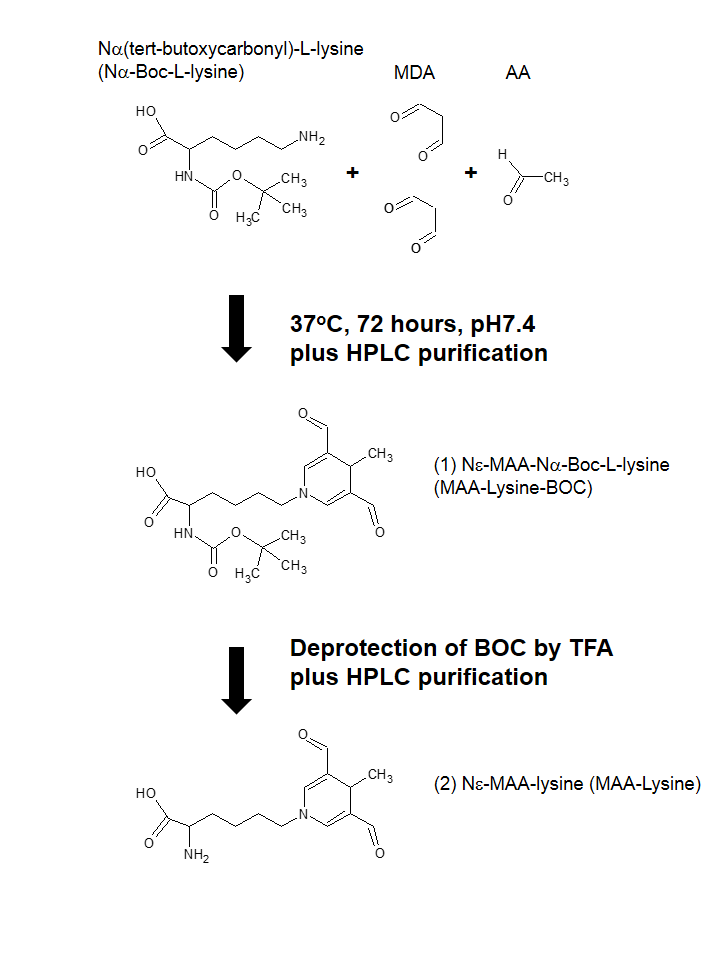
**

MAA-lysine preparation. Boc-lysine, acetaldehyde, and MDA were incubated at 37^o^C for 3 days. MAA-Boc-lysine was purified by HPLC system. The collected fractions were evaporated and further incubated with TFA to remove the Boc protecting group. MAA-lysine was then purified by the HPLC system.

**Figure S1.**

**C.**

**
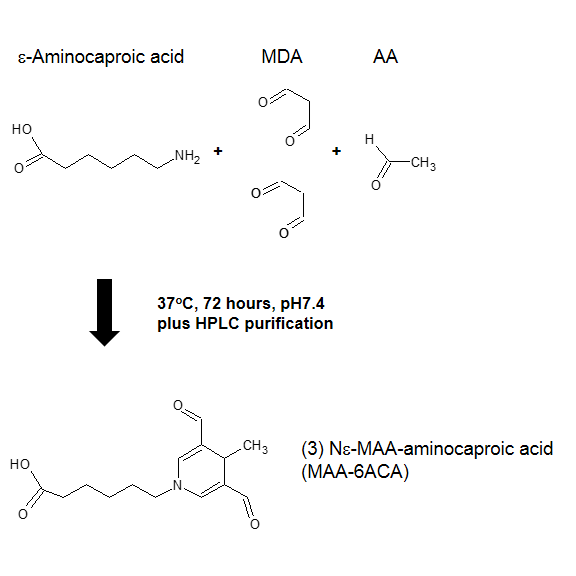
**

MAA-6ACA preparation. 6-ACA, acetaldehyde, and MDA were incubated at 37^o^C for 3 days. MAA-6ACA was purified by HPLC.
