# Supplementary material for: A purified MAA-based ELISA is a useful tool for determining anti-MAA antibody titer with high sensitivity
Source: PLoS One. 2017 Feb 21;12(2):e0172172. doi: 10.1371/journal.pone.0172172 (PMC5319763; doi:10.1371/journal.pone.0172172)
Supplement: S2 Fig — (DOCX) [file pone.0172172.s003.docx]

**Figure S.**

**A.**

**
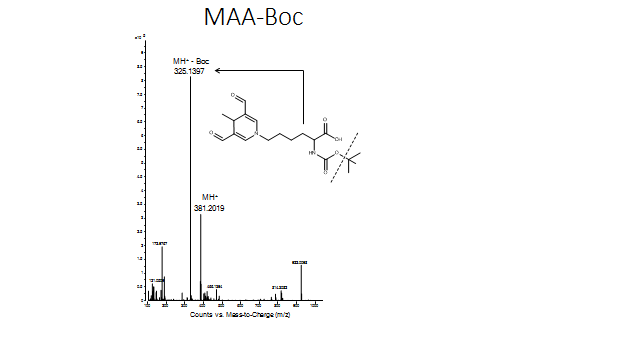
**

The full scan mass spectrum shows the protonated molecular ion of MAA-Boc-lysine at *m/z* 381.2019. Facile loss of the butyl group results in the major ion at *m/z* 325.1397. Other peaks are minor background ions and reference ions *m/z* 121.0509 and *m/z* 922.0098.

**Figure S2.**

**B.**

**H_2_O**

**a**

**b**

**i**

**e**

**c**

**d**

**f,g,h**

**k**

**j**

**DMSO**

^1^H NMR (DMSO-*d*_6_, 400 MHz) *S*-2((*t*-butoxycarbonyl)amino)-6(3,5-diformyl-4-methylpyridin-1(4*H*)-yl hexanioc acid (MAA-Boc-lysine).

**Figure S2.**

**C.**

**a**

**b**

**d**

**c**

**e**

**f,g,h**

**k**

**DMSO**

**H_2_O**

^1^H NMR (DMSO-*d*_6_, 400 MHz) *S*-2-amino-6-(3,5-diformyl-4-methypyridin-1(4*H*)-yl)hexanoic acid MAA-lysine)

**Figure S2.**

**D.**

**
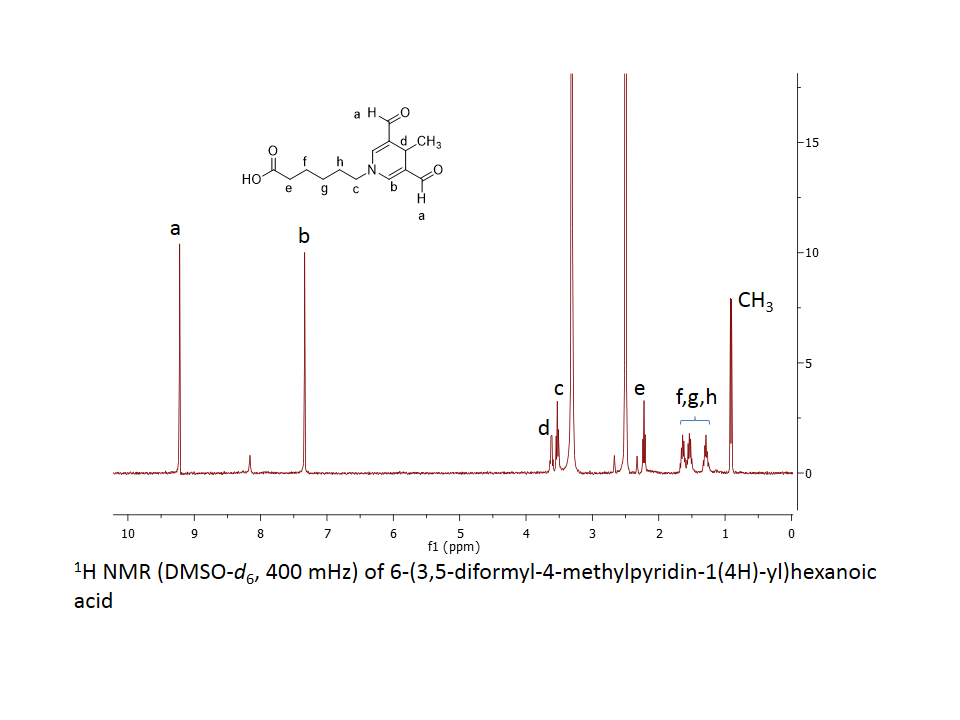
**
